# The Investigation of the Waveguiding Properties of Silk Fibroin from the Visible to Near-Infrared Spectrum

**DOI:** 10.3390/ma11010112

**Published:** 2018-01-11

**Authors:** Vaclav Prajzler, Kyungtaek Min, Sunghwan Kim, Pavla Nekvindova

**Affiliations:** 1 Department of Microelectronics, Faculty of Electrical Engineering, Czech Technical University, Technicka 2, 168 27 Prague, Czech Republic; 2 Department of Energy Systems Research, Ajou University, Suwon 16499, Korea; kyungtaekmin@gmail.com (K.M.); sunghwankim@ajou.ac.kr (S.K.); 3 Department of Physics, Ajou University, Suwon 16499, Korea; 4 Department of Inorganic Chemistry, Faculty of Chemical Technology, Institute of Chemical Technology, Technicka 5, 166 28 Prague, Czech Republic; pavla.nekvindova@vscht.cz

**Keywords:** silk fibroin, optical planar waveguides, optical losses

## Abstract

Silk fibroin protein has been reinvented as a new optical material for biophotonic applications because of its optical transparency, biocompatibility, and easy fabrication process. It is used in various silk-based optical devices, which makes it desirable to investigate the optical properties of silk from diverse perspectives. This paper presents our investigation of the optical properties of silk fibroin, extracted from *Bombyx mori* cocoons. We have measured transmission spectra from the visible to near-infrared region and investigated waveguiding properties by the prism-coupling technique for five wavelengths (473.0, 632.8, 964.0, 1311, and 1552 nm). From the measurements, we determined the values of refractive indices. The measurements also proved waveguiding properties for all of the wavelengths. Optical scattering losses were measured by the fiber probe technique at 632.8 nm and were estimated to be 0.22 dB·cm^−1^.

## 1. Introduction

Biocompatible optical materials have been intensively studied for the last few years as they offer many possibilities sensor applications. Nowadays, new photonic-based bio applications are sought among them as well. Especially biopolymers occurring in nature, such as silk fibroin, cellulose, and collagen, have been considered as attractive materials in bio-photonics for sensors, bio-optics, bio-micro-electromechanical systems (MEMS), imaging devices or therapeutics, optical waveguides, etc. [1,2,3]. One of these materials is *Bombyx mori* (silkworm) silk. Silkworm silk has already been widely used in the textile industry for thousands of years not only for its beauty but also because of its excellent physical and biomedical properties, such as flexibility, mechanical strength, biocompatibility, etc. [4]. All of these make the *Bombyx mori* silk an entirely new, unique material, with excellent future prospects. In particular, silk fibroin extracted from the *Bombyx mori* cocoon is promising as an optical biomaterial for bio-photonic applications [4,5]. 

Perhaps the most important parts of the photonic structures are optical waveguides, and many waveguide-based biosensors have already been developed [6]. The application of silk fibroin to such engineered structures is particularly suitable, which makes this material particularly interesting.

Self-assembled silk films and their crosslinked derivatives are optically transparent throughout the visible and near-infrared (NIR) regions, mechanically and chemically stable, and can be easily engineered to micron- and submicron-scale structures. Various kinds of silk fibroin-based optical devices have been developed, including photonic crystals [7], waveguides [4], distributed feedback lasers [8], metamaterials [9], and plasmonic structures [10,11]. For the design of all these optical devices, it is indispensable to characterize the optical properties of silk films, such as the refractive index and optical losses, precisely.

Regenerated silk fibroin has been entering the realm of biophotonics. However, despite all of its unquestionable benefits, the investigation of the basic optical properties of silk (regarding its utilization in photonics) has not been thorough enough to make it possible to design and demonstrate highly functional and complex optical components. In particular, it is very important to understand waveguiding properties precisely, because they are a cornerstone for the design of such optical components as laser cavities, interferometers, and photonic crystals. A silk optical waveguide has recently been presented [4], but the work did not provide any waveguiding properties in a broad spectral region, except for some information on the refractive index only for one wavelength, namely at 632.8 nm.

Information on the refractive indices of any photonic material in a broad spectral region is of particular importance for the precise design of highly sophisticated waveguiding structures. In our case, this concerns the refractive indices of the silk fibroin in visible and infrared spectra, but such data are still missing, which is why we have undertaken the presented study.

This paper aims to report on the refractive indices of the deposited silk thin film measured by the prism-coupling technique, which works on the principle of dark-mode spectroscopy. Our prism-coupling setup has allowed for the measurement of the waveguiding properties of the silk layer, including optical-loss measurement. The optical scattering losses of silk planar waveguides have been measured by the fiber scanning method at 632.8 nm (He-Ne laser). We have studied the waveguiding properties of the silk-fibroin layers at five wavelengths (473, 632.8, 964, 1311, and 1552 nm) and confirmed that the silk layers exhibit the waveguiding properties for all of the measured wavelengths with low optical losses of 0.22 dB·cm^−1^ at 632.8 nm.

## 2. The Preparation of the Samples

The preparation of an optical material from silk consists of two steps: first, a silk aqueous solution is prepared and then it is deposited on a glass or silica-on-silicon substrate in a form of a planar optical waveguide.

The preparation of a silk aqueous solution: *Bombyx mori* cocoons were boiled in a solution of 0.02 M Na_2_CO_3_ for 60 min to remove sericin. The remaining fibroin was rinsed with distilled water and then air-dried for 24 h. Subsequently, the fibroin was dissolved in a 9.3 M LiBr solution at 60 °C for 4 h, yielding a 20 wt % aqueous solution. For two days, the silk/LiBr solution was dialyzed against distilled water using a dialysis cassette (Slide-a-Lyzwe, Pierce, MWCO 3.5 K, ThermoFisher Scientific, Waltham, MA, USA) at room temperature. The dialyzed solution had a concentration of 6%. The obtained solution was purified using a centrifuge, and a syringe filter with a pore size of 0.45 μm.

The fabrication of silk planar waveguides: A silk aqueous solution with a concentration of 6% was spin-coated at 2000 rpm on borosilicate Glass D263 (Shott) or on a silica-on-silicon (Si/SiO_2_) substrate. A silk layer with a thickness of about 1068 nm on a Si/SiO_2_ substrate and another approximately 820 nm thick on borosilicate glass were fabricated by single spin-coating. For the formation of thicker silk films, spin coating was performed multiple times. Before each step of additional coating, the previously formed silk films were dipped in methanol for 3 min and dried at 60 °C to produce a water-insoluble silk film by a beta-sheet conformation [12].

## 3. The Modelling/Design of Silk Optical Planar Waveguides

**Figure 1:**
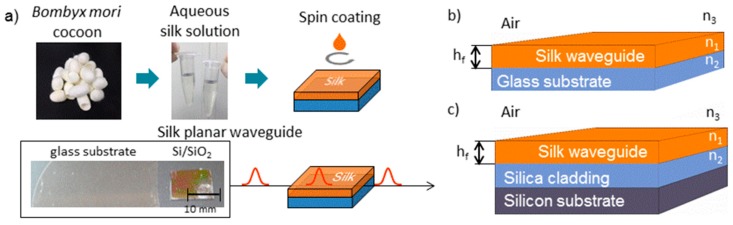
(**a**) A schematic view of the silk optical planar waveguide deposition procedure; (**b**) silk optical planar waveguide on glass substrate; (**c**) silk optical planar waveguide on silica-on-silicon substrate.

The fabrication of silk films and the designed silk waveguide structure are schematically depicted in Figure 1. Figure 1a shows the fabrication of thin silk films, Figure 1b shows a silk planar optical waveguide deposited on a glass substrate, and Figure 1c shows a silk waveguide structure deposited on a silicon substrate with silica cladding layers. In this case, the silk waveguides are asymmetrical as they consist of a waveguide layer with the index of refraction of the core waveguide *n*_1_ (silk) and lower refractive indices of the substrate *n*_2_ (silica-on-silicon or glass) and of the cover layer *n*_3_. In this case, the upper layer is the air.

Borosilicate glass and silica-on-silicon substrates were considered in the calculations as the substrates onto which silk films were deposited. In the waveguiding layers, a standing wave will be formed based on the principle of transverse resonance; it is possible to derive a dispersion Equation (1) from it [13,14]:

(1) $$\frac{2\cdot \pi }{\lambda_{0}}\cdot h\cdot \sqrt{n_{1-}^{2}n_{eff}^{2}}=arctan\left(p_{12}\cdot \sqrt{\frac{n_{eff}^{2}-n_{2}^{2}}{n_{1}^{2}-n_{eff}^{2}}}\right)+arctan\left(p_{13}\cdot \sqrt{\frac{n_{eff}^{2}-n_{3}^{2}}{n_{1}^{2}-n_{eff}^{2}}}\right)+k\cdot \pi$$

where *λ*_0_ is vacuum wavelength, h is the thickness of the planar waveguide, *n*_1_ is the refractive index of the core layer (in our case silk layer), *n*_2_ is the refractive index of the substrate (in our case Si/SiO_2_ or glass), *n*_3_ is the refractive index of the cover layer (in our case air), *n_eff_* is the effective refractive index of the optical planar waveguide, and *k* is an integer number—*k* = 0, 1, 2 … The *p*_12_ and *p*_13_ are defined for the transverse electric polarized light (TE) modes:

(2) $$p_{12}=p_{13}=1$$

and for the transverse magnetic polarized light (TM) mode as:

(3) $$p_{12}={\left(\frac{n_{1}}{n_{2}}\right)}^{2},\;p_{13}={\left(\frac{n_{1}}{n_{3}}\right)}^{2}$$

From the solution of the dispersion Equation (1), it is possible to estimate the thickness *h_f_* for single-mode operation optical planar waveguides [14,15]:

(4) $$h_{f}\;=\frac{\lambda_{0}}{2\cdot \pi \cdot \sqrt{n_{1}^{2}-n_{2}^{2}}}\left(k\cdot \pi +\arctan\left(p_{13}\sqrt{\frac{n_{2}^{2}-n_{3}^{2}}{n_{1}^{2}-n_{2}^{2}}}\right)\right).$$

**Figure 2:**
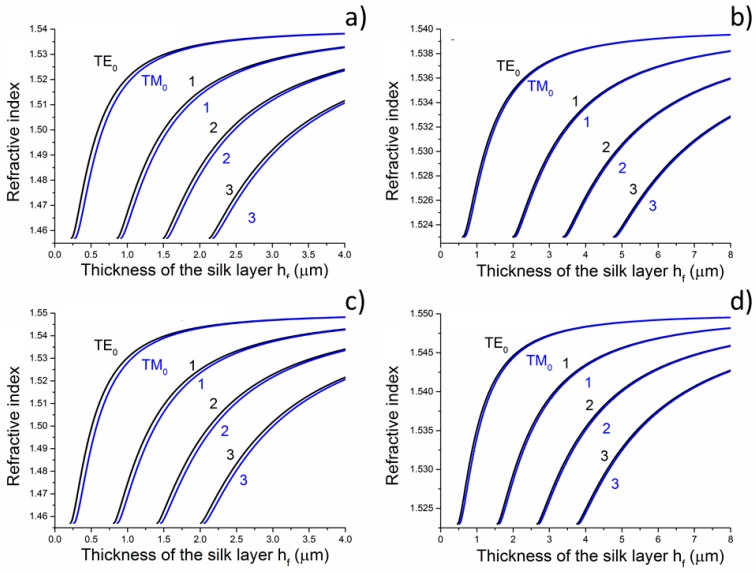
Mode calculations performed for the wavelength of 632.8 nm of the silk optical planar waveguides: (**a**) silk layer (*n_f_* = 1.540) deposited on a Si/SiO_2_ substrate; (**b**) silk layer (*n_f_* = 1.540) deposited on a borosilicate glass substrate; (**c**) silk layer (*n_f_* = 1.550) deposited on Si/SiO_2_; and, (**d**) silk layer (*n_f_* = 1.550) deposited on a borosilicate glass substrate.

Before the deposition, the dimensions of the silk waveguides were designed utilizing the refractive-index values listed in Table 1 and the results are shown in Figure 2. The refractive indices that were used for modelling for silk materials were published in [4,16], for Si/SiO_2_ substrate in [17] and for borosilicate glass substrate in [18].

For the waveguide structure described above, the results of mode calculations performed for an operating wavelength of 632.8 nm for TE as well TM polarization for silk planar waveguides deposited on a silica-on-silicon substrate are shown in Figure 2a, and the calculation for silk waveguides that are deposited onto borosilicate glass is shown in Figure 2b. Both figures show the results of the calculations based on the silk refractive index of 1.540. Figure 2c,d show such calculations based on the silk refractive index of 1.550 (see Table 1).

It arises from the calculations, for example, that if we want the deposited silk layer (*n_f_* = 1.540) to guide one single TE mode onto a silica-on-silicon substrate, the thickness of the silk layer should be between 228 nm and 863 nm—see Figure 2a and Table 1. Alternatively, for a single-mode silk waveguide (*n_f_* = 1.540) deposited onto borosilicate glass, the thickness of the silk layer should range from 607 nm to 1993 nm for the TE mode—for more details, see Figure 2b. Table 1 also shows critical thickness *h_f_* if we assume refractive index of silk layer *n_f_* = 1.550.

## 4. The Properties of the Silk Layer Waveguides

### 4.1. Surface Morphology

For experimental investigation into waveguiding properties, it is of enormous importance to generate a uniform and smooth waveguide layer in the fabrication process because even nanoscale bumps and cracks can scatter the confined light, which is undesirable for the investigation into the optical traits of materials. Therefore, we have examined the surface morphology of the coated silk surface using atomic force microscopy (AFM, Ntegra Spectra instrument, Zelenograd, Russia), performed on an NT-MDT Ntegra Spectra instrument (Ntegra Spectra instrument, Zelenograd, Russia) in a tapping (semicontact) mode at room temperature. The layer of silk was measured on four samples, two of which were Si/SiO_2_ substrates and two borosilicate glass substrates. The measured data were assessed using ten point heights, which involved the evaluation of the difference between the mean value of the heights of the five highest peaks and the mean value of the five lowest valleys along the assessment lengths. The results are graphically illustrated in Table 2 and show that no significant differences have been found between the measured surfaces of the two kinds of samples. The first column of Table 2 includes the sample 1 area of 25 × 25 µm^2^ together with a detail of a selected area between the peaks described above. The second columns show the same images for sample 3. For all of the images, the R values have been calculated. These results clearly evidence that only a few of the observed protrusions are not bigger than 300 nm and do not reveal any structure, so that they are likely to be some particles of dust stacked to the sample surfaces. At this moment, we are not able to conclude whether the protrusions have arisen from the process or not.

### 4.2. Raman Spectra

**Figure 3:**
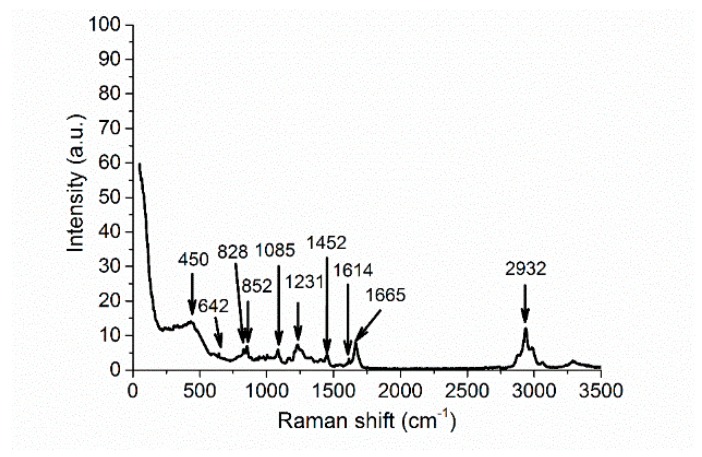
The Raman spectra of the silk sample deposited on a silica-on-silicon substrate (silk #1).

Raman analysis was performed on a DXR Raman Microscope spectrometer of the company Thermo Scientific (Edison, NJ, USA) equipped with a confocal Olympus microscope (Edison, NJ, USA). A solid-state Nd:YAG laser (wavelength: 532 nm, maximum power: 10 mW) was used as an excitation source. The measurement conditions included the power of 2–8 mW, 10 accumulations of 10 s scans, a grating with 900 lines/mm, and the aperture being a 25 µm pinhole. Detection was performed using a multichannel thermoelectrically cooled CCD camera (Edison, NJ, USA). 5× magnification provided the measurement spot-size of ~1 µm^2^. The resulting RAMAN spectrum was obtained after subtracting spectra from the Si/SiO_2_ substrate standard (see Figure 3). 

Silk fibroins exhibit characteristic conformational bands in the ranges of 1650–1667 cm^−1^ and 1241–1279 cm^−1^, which correspond to amide I and complex amide III, respectively [19,20]. The amide I (random coil) pristine band was observed at 1665 cm^−1^ and the amide III (β-sheet) pristine band at 1231 cm^−1^. The arrows mark the Raman-active bands at 1085 cm^−1^, 1232 cm^−1^, and 1667 cm^−1^, which are characteristic of the main proteins in the partially crystallized silk, indicating that the silk film is mechanically and chemically stable under ambient conditions; the remaining features of the spectrum are very similar to the spectrum published in [3,21]. We have also observed bands at 450, 642 (644), 828 (830), 852 (854), 1003 (1004), 1452 (1455), and 1614 (1616) cm^−1^, attributable to silk fibroins [20]. The 2932 cm^−1^ bands correspond to aliphatic C-H bonds in the silk structure. 

### 4.3. Transmission Spectra/UV-VIS Spectroscopy

**Figure 4:**
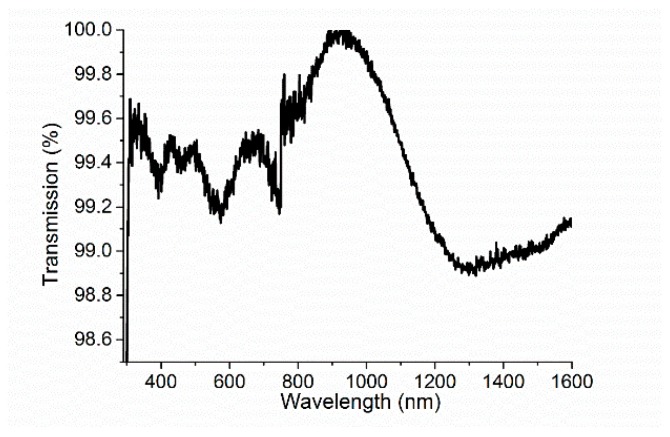
The transmission spectra of silk deposited on a glass substrate (silk #2).

The transmission spectra of the silk film deposited onto borosilicate glass with a thickness of about 820 nm were collected by a UV-VIS-NIR spectrometer (UV-3600 Shimadzu, Shimadzu Deutschland GmbH, Duisburg, Germany) in the spectral range of 300–1600 nm; they are given in Figure 4. The figure has proven that silk is transparent in the visible range for near-infrared light and transmission spectra show that the transmission occurs in the range of 99.8–99.9%. Our results are in good agreement with [22].

### 4.4. Refractive Index

**Figure 5:**
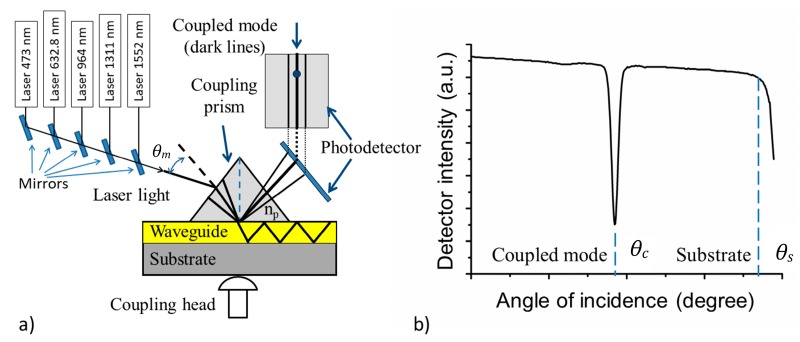
(**a**) A schematic view of the prism-coupling technique; (**b**) the assessment of the critical angle of incidence *θ_c_* (coupled mode) and *θ_S_* (substrate), used for the evaluation of the refractive index of the guiding mode for planar optical waveguides [27].

Effective refractive indices were determined by the prism-coupling technique [23], also known as dark-mode spectroscopy or m-lines spectroscopy. The main advantage of this technique is the assessment of the waveguiding properties of the thin films (the number of modes, refractive index value, and optical losses). The measurement was performed using a Metricon model 2010/M prism coupler (Metricon Corporation, Pennington, NJ, USA) [24] at room temperature for five wavelengths (473, 632.8, 964, 1311, and 1552 nm) for transverse electric (TE) polarizations. For optical coupling, we used the coupling prism #200-P-4a (*n*(measuring range) = 1.2–2.02, *λ* = 633 nm (prism code: 6600, refractive index: 2.1558 at 632.8 nm)). The optical contact between the prism and the measured silk samples was created by applying pressure on the coupling head without using immersion gel. A schematic view of the measurement setup is shown in Figure 5a [25]. The index of refraction of a planar optical waveguide can be determined by measuring the critical angle of incidence *θ_m_* (see Figure 5b). The laser beam is coupled into a planar waveguide via a coupling prism (the refractive index *n_p_* and angle *A_p_*) and it is governed by the angle of incidence *θ_m_* of the light impinging onto the prism base. This angle *θ_m_* determines the phase velocity *v_i_* of the incident wave in the coupling prism and in the air gap:

(5) $$\nu_{i}=\frac{c}{n_{p}}\cdot \sin(\theta_{m})$$

The coupling of laser light into the planar waveguide only occurs when *θ_m_* is selected, such that the phase velocity *v_i_* matches the phase velocity *v_m_* of one of the modes of propagation in the waveguide (*m* = 0, 1, 2, …). By determining the angles of resonance *θ_m_*, we can experimentally find the effective indices *N_m_* of mode m from the relations [26]:

(6) $$N_{m}=\frac{c}{\nu_{m}}=n_{p}\cdot \sin(\theta_{m})$$

(7) $$\theta_{m}=A_{p}+\arcsin(\frac{\sin(\theta_{m})}{n_{p}})$$

When a guided mode is excited, the TE or TM mode appears as a sharp line on the reflected intensity spectrum. From the angular position of the guided modes, one can thus calculate the corresponding refractive index. For our measurement, the routine refractive index resolution is ±0.0003, and the absolute accuracy is ±0.0005.

**Figure 6:**
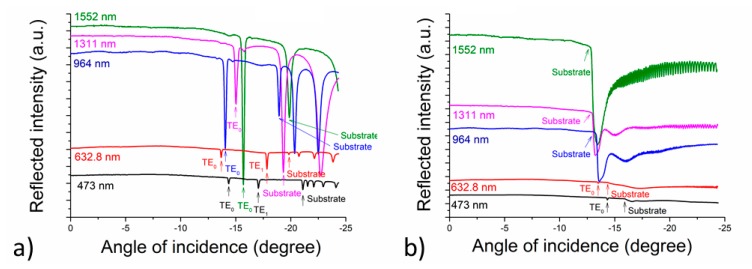
The assessment of the critical angles of incidence *θ_c_* used for the evaluation of the refractive indices of the silk deposited onto: (**a**) a silica-on-silicon substrate (silk #1); and (**b**) a borosilicate glass substrate (silk #2).

An example of the assessment of the silk refractive index (i.e., the dependence of the detector intensity on the angles of incidence *θ*) is shown in Figure 6.

Figure 6a shows the angles of incidence of the silk samples deposited on Si/SiO_2_ and Figure 6b shows the results for silk samples deposited on borosilicate glass. From the measurement, it is possible to determine not only the refractive indices of the planar waveguides, but also the refractive indices of the substrates used—see Figure 6b [27,28]. From the results shown in Figure 6 and summarized in Table 3, it is clearly evident that the silk layers exhibit waveguiding properties. The refractive indices of the deposited films were calculated using the angles given in Figure 6a for silk layers deposited onto a silica-on-silicon substrate and Figure 6b for silk layers deposited onto a glass substrate having a thickness of 1.078 μm. It may be deduced from Figure 6 and Table 3 that the silk planar waveguide deposited on Si/SiO_2_ guides two TE modes at the wavelengths of 473 and 632.8 nm and one TE mode at 964, 1311, and 1552 nm. The silk planar waveguide deposited on borosilicate glass guides one mode at 473 and 632.8 nm; no modes at longer wavelengths have been found.

**Figure 7:**
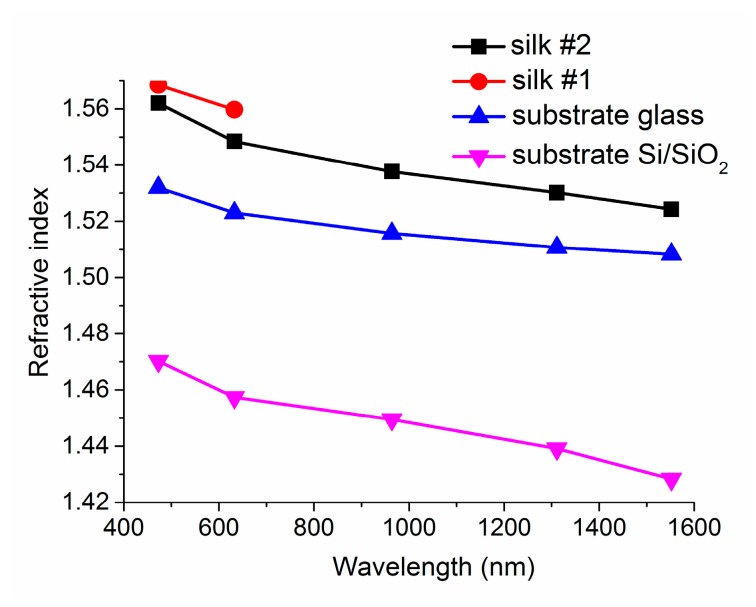
The refractive indices of silk planar optical waveguides deposited on silica-on-silicon (silk #1) and glass substrates (silk #2).

The calculated refractive indices for the five wavelengths from Figure 6 are shown in Figure 7. It arises from the figure that at the wavelengths of 472 and 632.8 nm, the values of the refractive indices are almost the same for silk deposited both on silica-on-silicon and glass substrates. We were not able to measure the refractive-index values for longer wavelengths (964, 1311, and 1552 nm) as the silk layers were not thick enough to provide light to pass through them. Generally, the numbers of supporting modes corresponded well with the expectation from the calculations, according to Equation (4).

In comparison with the results published by other authors (Ref. [4]), our refractive indices are slightly higher (1.5481 for the silk deposited on a silica-on-silicon substrate and 1.5507 for the silk deposited on glass in contrast with their 1.54 at 633 nm). The authors do not mention any other refractive indices for any other wavelengths. The value of the refractive index given in Ref. [16] is 1.55, without any specification of the method or wavelength used.

The refractive index of spider silk (orb-weaver spiders, Araneidae keyserlingi) is 1.54–1.58 at 589 nm, or 1.50 in the case of native spider silk ([29] and the references therein).

### 4.5. Waveguide-Loss Measurement

**Figure 8:**
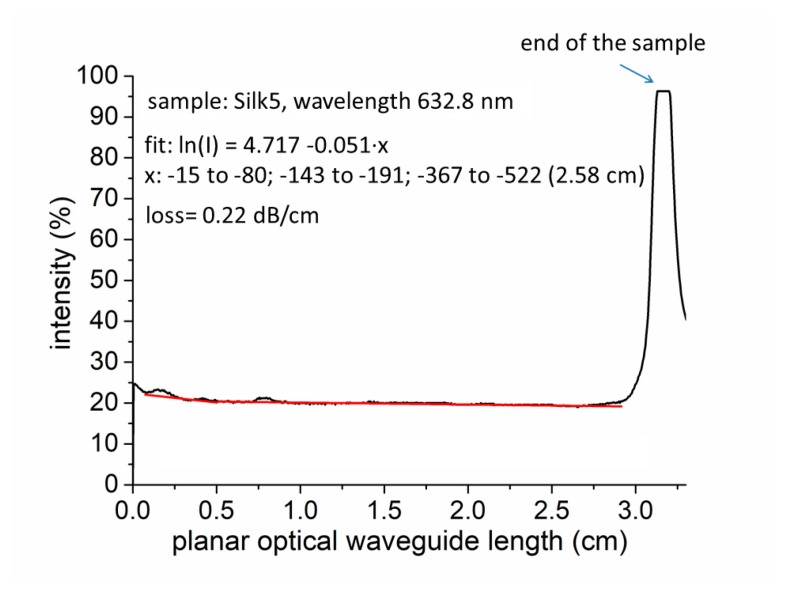
The optical losses of the silk deposited on a glass substrate (silk #2).

The optical scattering losses of the planar waveguides were measured by the fiber scanning method [30,31,32]. This method measures the losses of optical waveguides by scanning a fiber optic probe and photodetector down the length of a propagating streak to measure the light intensity scattered from the surface of the guide. The assumption is that at every point of the propagating streak, the light scattered from the surface and collected by the fiber is proportional to the light that remains within the guide. The best exponential fit to the resulting intensity vs. distance curve yields the loss in dB·cm^−1^. The results of the optical-loss measurements for a silk sample deposited onto a Si/SiO_2_ substrate are provided in Figure 8. Our optical silk planar waveguides had optical losses as low as 0.22 dB·cm^−1^ at 632.8 nm.

Optical losses were lower than those reported in [4]—0.25 dB·cm^−1^ (632.8 nm, He-Ne laser), which were measured for a channel of 5 × 5 µm^2^ silk waveguides fabricated by printing silk using an ultra-dispensing system. The losses measured by the authors were 0.25 dB·cm^−1^ and 0.81 dB·cm^−1^ for straight and curved waveguides, respectively. Their losses obtained by propagating light in 820-mm-thick silk fibroin films yielded values between 0.25 dB·cm^−1^ and 0.75 dB·cm^−1^. Note, however, that our losses were measured in a planar waveguide.

Spider silk fiber waveguides presented in [33] have optical losses around 10.5 dB·cm^−1^, i.e., much higher than the optical losses of our planar silk waveguide—0.22 dB·cm^−1^.

## 5. Conclusions

The paper reports on the waveguide properties of silk fibroin optical planar waveguides, extracted from *Bombyx mori* cocoons in terms of their possible utilization in photonic structures operating in the visible and infrared region. Such knowledge is of particular interest for the design of sophisticated structures for photonics and sensor applications. Raman spectroscopy was used to characterize the molecular structures of silk fibroin with three typical bands being observed at 1085 cm^−1^, 1232 cm^−1^, and 1667 cm^−1^, the signatures of the mechanically and chemically stable silk waveguide layer. The transmission spectra of the silk film deposited onto borosilicate glass have proven that the silk film is highly transparent in the visible and near-infrared region. The prism-coupling technique has been used to obtain the values of refractive indices for five wavelengths (473, 632.8, 964, 1311, and 1552 nm). Silk layers have revealed waveguiding properties for all of the measured wavelengths, including the infrared ones. This makes it possible to utilize them also in the so far rarely exploited infrared region, which concerns bio-materials in photonics. This is further supported by the fact that this is a low-loss material (our best sample has optical losses as low as 0.22 dB·cm^−1^ at 632.8 nm). Our study provides the accuracy and reliability for the future design and demonstration of those promising silk bio-optical components that are suitable for many applications, including bio-sensing and similar functions.

## Figures and Tables

**Table 1:** Refractive indices used for design of the silk planar waveguides for wavelength 632.8 nm and calculation of thickness *h_f_* for supporting transverse electric (TE)_0_ and TE_1_ modes.

-		**Refractive Index**	
silk layer waveguide—*n*_1_		1.540 [2]	1.550 [15]
Si/SiO_2_ substrate—*n*_2_	1.457 [16]	1.457 [16]	
glass substrate—*n*_2_	1.523 [17]	1.523 [17]	
**-**	***h_f_*** **Thickness (μm)**		
TE_0—_Si/SiO_2_/silk/air	0.228 *	0.211 **	
TE_1—_Si/SiO_2_/silk/air	0.863 *	0.809 **	
TE_0—_glass/silk/air	0.607 *	0.463 **	
TE_1—_glass/silk/air	1.993 *	1.562 **	

* *n_f_* = 1.540, ** *n_f_* = 1.550.

**Table 2:** Atomic force microscopy (AFM) measurement of the silk layer deposited on Si/SiO_2_ (sample 1) and on borosilicate glass substrates (sample 3).

**Sample**	**Sample 1 (Si/SiO_2_/Silk)**	**Sample 3 (Glass/Silk)**
area	2510 µm^2^	2501 µm^2^
R_pv_ (nm)	184 nm	141 nm
R_z_ (nm)	90 nm	58 nm
R_a_ (nm)	1.7 nm	2.2 nm
R_q_ (nm)	4.9 nm	3.8 nm
3D pictures	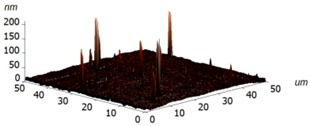	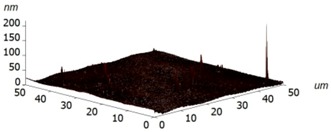
**Area without Peaks**		
area	258 µm^2^	318 µm^2^
R_pv_ (nm)	6 nm	7 nm
R_z_ (nm)	3.2 nm	3.2 nm
R_a_ (nm)	0.7 nm	0.6 nm
R_q_ (nm)	1.0 nm	0.8 nm
3D pictures	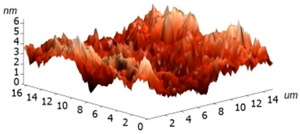	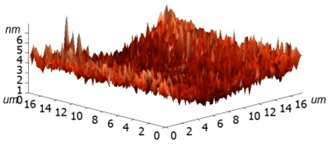

R_pv_—peak-to-valley, R_z_—ten-point height, R_q_—root-mean-square roughness, R_a_—the average roughness.

**Table 3:** The evaluation of silk refractive indices. The waveguiding properties of the deposited silk layer: the angle of incidence of the TE modes.

Wavelength (nm)	Silk #1 (Si/SiO_2_)		Silk #2 (Borosilicate Glass)	
	*θ_c_* (Degree)	*n*	*θ_c_* (Degree)	*n*
473 mode TE_0_	−14°30′	1.5511	−14°20′	1.5512
473 mode TE_1_	−17°03′	1.5184	not observed	
632.8 mode TE_0_	−13°41′	1.5307	−13°29′	1.5329
632.8 mode TE_1_	−16°20′	1.4804	not observed	
964 mode TE_0_	−14°03′	1.5058	not observed	
1311 mode TE0	−15°00′	1.4852	not observed	
1552 mode TE0	−15°42′	1.4733	not observed	

*θ_c_*—the angle of incidence, *n*—the refractive index of the silk layer.
